# Assessing Breastfeeding Attitudes and Self‐Efficacy Among Health care Personnel and Women With Multiple Sclerosis: Two Cross‐Sectional Surveys

**DOI:** 10.1002/brb3.70468

**Published:** 2025-04-18

**Authors:** Solange M. Saxby, Carlyn Haas, Anna Klein, Tyler J Titcomb, Farnoosh Shemirani, Terry Wahls, Linda Snetselaar, Christine Gill, Pamela Mulder

**Affiliations:** ^1^ Department of Internal Medicine University of Iowa Iowa City Iowa USA; ^2^ Department of Community and Family Medicine, Dartmouth Health Lebanon New Hampshire USA; ^3^ Epidemiology Department University of Iowa Iowa City Iowa USA; ^4^ Department of Neurology University of Iowa Hospital and Clinics Iowa City Iowa USA; ^5^ College of Nursing University of Iowa Iowa City Iowa USA

**Keywords:** attitudes, breastfeeding, multiple sclerosis, self‐efficacy

## Abstract

**Introduction:**

This study aimed to describe breastfeeding attitudes among health care personnel, as well as breastfeeding attitudes and self‐efficacy in women with multiple sclerosis (MS), who are currently or have previously breastfed.

**Methods:**

Two cross‐sectional surveys were sent electronically to health care personnel at a single center to capture attitudes toward breastfeeding in women with MS using the Iowa Infant Feeding Attitudes Scale (IIFAS), and women with MS who were currently or had previously breastfed to measure breastfeeding attitudes and self‐efficacy using the IIFAS and Breastfeeding Self‐Efficacy Survey‐Short Form (BSES‐SF). Descriptive statistics and one‐way analysis of variance were used to assess differences among categories of participant demographics.

**Results:**

In the health care personnel survey, among health care specialties, neonatology exhibited the highest mean scores on the IIFAS (69.8 ± 8.89), reflecting positive attitudes, while neurology and students had the lowest mean IIFAS scores (62.4 ± 10.3 and 58.2 ± 3.94, respectively) with neutral attitudes. Health care personnel with 16 or more years of service demonstrated positive attitudes toward breastfeeding (70.9 ± 9.30), as assessed by IIFAS. In the survey of women with MS, women identifying as Middle Eastern/North African had the highest mean IIFAS score (78.0 ± 5.66), indicating positive breastfeeding attitudes, while women identifying as Black had the lowest (62.7 ± 6.07), reflecting a neutral attitude. Positive attitudes were revealed by participants who exclusively breastfed for 6 months (70.1 ± 7.17) and who had three or more children (70.1 ± 6.17). Participants who exclusively breastfed for 6 months and who had breastfed three or more children demonstrated the highest breastfeeding self‐efficacy as assessed by BSES‐SF scores (52.2 ± 4.93 and 51.7 ± 5.26, respectively).

**Discussion:**

Attitudes towards breastfeeding in women with MS differed by health care specialty and years of service in health care personnel. Among women with MS, infant feeding attitudes and breastfeeding self‐efficacy varied based on ethnicity, age, number of children, number of children breastfed, and breastfeeding exclusivity.

## Graphical abstract

AIM: Describe breastfeeding attitudes among healthcare personnel, as well as breastfeeding attitudes and self‐efficacy in women with multiple sclerosis (MS), who are currently or have previously breastfed.

RESULTS: SURVEY 1: Among healthcare specialties, neonatology exhibited positive attitudes towards breastfeeding, while neurology and students had neutral attitudes towards breastfeeding. Healthcare personnel with 16 or more years of service demonstrated positive attitudes toward breastfeeding.

SURVEY 2: Positive attitudes were revealed by women with multiple sclerosiswho exclusively breastfed for six months, and who had 3 or more children. Women with multiple sclerosis who exclusively breastfed for six months and who had breastfed 3 or more children demonstrated the highest breastfeeding self‐efficacy.

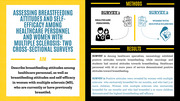

## 1 Introduction

Multiple sclerosis (MS) is a chronic immune‐mediated neuroinflammatory condition increasing in global prevalence (Walton et al. 2020). In the past 30 years, MS has disproportionately affected females, who have a three‐fold increased risk compared to males (Varytė et al. 2020). The onset of MS is common during the third and fourth decades of life, which coincides with a woman's childbearing years (Varytė et al. 2020), and recent studies suggest up to one‐third of patients of reproductive age have become pregnant after disease onset (Graham et al. 2024; Moehlman et al. 2024). The risk of relapse has been shown to be decreased during pregnancy; however, there is concern for a higher risk of relapse in the first 6 months postpartum (Confavreux et al. 1998). These first 6 months are vital during a newborn's life, during which the World Health Organization, the American Academy of Pediatrics, and the American Academy of Obstetricians and Gynecologists all recommend exclusive breastfeeding (Meek and Noble 2022; American College of Obstetricians and Gynecologists 2021). Women consider multiple factors when deciding against exclusive breastfeeding, including breast discomfort, concerns about milk production, and socioeconomic and work‐related constraints (Feenstra et al. 2018; Aigner et al. 2023); however, the factors contributing to deciding against exclusive or any breastfeeding for 6 months among women with MS are not well understood.

The majority of women with MS worry about their ability to breastfeed due to their use of disease‐modifying therapies (DMTs) (Peper et al. 2023). Emerging evidence indicates that some DMTs are compatible with breastfeeding while others are contraindicated, though further research is still required to assess the associated risks (Graham et al. 2024). As a result, clear clinical guidelines on restarting DMTs after childbirth are still lacking, leaving women with MS apprehensive about the safety of medication use during breastfeeding (Graham et al. 2024). Recent recommendations encourage most postpartum women with MS to breastfeed exclusively for 6 months and continue breastfeeding afterward per their preference (A. M. Langer‐Gould 2019). A meta‐analysis showed as much as a 43% reduction in the risk of relapse with exclusive breastfeeding for at least 2 months postpartum compared to partial breastfeeding (Krysko et al. 2020). Furthermore, recent evidence suggests a decreased incidence in relapse rate for 10–12 months postpartum, possibly attributable to periconceptional DMTs and exclusive breastfeeding (Schubert et al. 2023). Women with MS continue to make the difficult choice to forgo breastfeeding and resume DMTs or, alternatively, breastfeed and live untreated; these choices, largely influenced by fears of postpartum relapses, greatly affect family planning, treatment, and breastfeeding choices for women with MS (A. Langer‐Gould et al. 2020; Almas et al. 2016).

When looking for information about pregnancy and motherhood with MS, most women reach out to general neurologists, MS specialists, obstetrician–gynecologists, and/or primary care physicians (Peper et al. 2023; Pebdani et al. 2015); although, less than half of women with MS report receiving any counseling about breastfeeding (Aigner et al. 2023; Pebdani et al. 2015). Women with MS who see multiple providers may receive conflicting information about breastfeeding that makes them feel less safe, with some women reporting negative reactions and criticism from their physicians about their desire to have children (Özkan and Polat Dünya 2023). Breastfeeding attitudes of health care professionals significantly influence the kind of breastfeeding support they offer to mothers, which, in turn, can influence women's attitudes and decisions around breastfeeding (Ekström et al. 2005), as well as breastfeeding self‐efficacy (Meedya et al. 2010), which is a mother's perceived ability to breastfeed her newborn (Dennis 1999). Therefore, this study aims to describe the breastfeeding attitudes of health care personnel towards women with MS, as well as the breastfeeding attitudes and self‐efficacy of women with MS.

## 2 Methods

### 2.1 Study Design and Setting

Two cross‐sectional quantitative surveys were designed to assess the breastfeeding attitudes of health care personnel for women with MS and breastfeeding attitudes and self‐efficacy among women with MS. Therefore, surveys were sent to two target populations: (1) health care personnel who work with women with MS (the Bf MoMS HP ‐ Survey 1) and (2) women diagnosed with MS who have previously breastfed (the Bf MoMS PP ‐ Survey 2). However, the original aim was also to collect responses from health care personnel across the United States; although a low response rate to the survey led to a discontinuation of the survey after 3 months, with a focus on convenience sampling of health care personnel from the author's single institution. All study protocols and procedures were followed in accordance with the ethical standards from the Helsinki Declaration and were reviewed and approved by the University of Iowa Institutional Review Board (IRB# 202305124) prior to the study commencing. Additionally, in adherence to best practices for reporting cross‐sectional studies, this manuscript was prepared in accordance with the Checklist for Reporting of Survey Studies (CROSS) guidelines (Sharma et al. 2021).

### 2.2 Survey Development and Pretesting

Two cross‐sectional quantitative surveys, Bf MoMS HP—Survey 1 and Bf MoMS PP—Survey 2, were developed at the University of Iowa Hospitals and Clinic (UIHC; Iowa City, Iowa), a Level 1 Trauma Center for Pediatrics, Level 4 NICU, and one of the MS Neurological Centers in Iowa. The two surveys were formatted electronically through Research Electronic Data Capture (REDCap), a secure, web‐based software platform designed to support data capture for research studies (Harris et al. 2019; Harris et al. 2009).

The Bf MoMS HP—Survey 1 consisted of 59 total items, made up of the demographics questionnaire and two validated questionnaires, the Iowa Infant Feeding Attitudes Scale (IIFAS) and the Breastfeeding Attitudes Scale (BAS), which have been previously used to assess attitudes about breastfeeding among medical personnel in a community hospital (Quinn and Tanis 2020). Participants were instructed to answer the IIFAS and BAS in the context of breastfeeding for women with MS. To ensure and maintain the anonymity of participants, no names or contact information were collected in the Bf MoMS HP—Survey 1. Demographic data included sex (male, female, or other); profession (physician, fellow/resident physician, physician associate, nurse, nurse practitioner, medical assistant, medical student, nursing student, or other); medical specialty (neurology, obstetrics, pediatrics, neonatology, family medicine, current health sciences student, or other); and years of service (student, 0–5 years, 6–10 years, 11–15 years, and 16+ years).

The Bf MoMS PP—Survey 2 consisted of 54 items, including the demographics questionnaire and two validated questionnaires, the IIFAS and the Breastfeeding Self‐Efficacy Survey—Short Form (BSES‐SF), which have been widely used to evaluate postpartum mothers' infant feeding attitudes and breastfeeding self‐efficacy, respectively (Amini et al. 2019; Mitchell‐Box et al. 2013; Gizaw et al. 2022). The demographic data collected, included: date of birth, age, sex (male, female, other), country of residence (USA, International), MS diagnosis type (clinically isolated syndrome, relapse‐remitting MS (RRMS), primary progressive MS (PPMS), secondary progressive MS (SPMS), other), MS diagnosis date, DMTs taken during breastfeeding (yes or no), marital status (married, single, divorced, widowed, domestic partnership, and other), race (American Indian or Alaska Native, Asian, Hispanic or Latino, Middle Eastern/North African, Native Hawaii or Other Pacific Islander, White, Other, or Prefer not to answer), employment status (full‐time, part‐time, self‐employment, unemployed/homemaker, student, or other), the total number of children (1, 2, and 3+), number of children breastfed exclusively for 6 months (1, 2, and 3+), and the total number of children breastfed for any time or amount (1, 2, and 3+).

Developed by Mora et al. 1999, the IIFAS is a validated and reliable survey instrument to measure maternal attitudes toward infant feeding methods. The instrument contains questions that indicate the extent to which respondents agree with each statement on a 5‐point Likert‐type scale ranging from 1 (*strongly disagree*) to 5 (*strongly agree*). Each question is added together for a total IIFAS score that can range from 17 to 85, with higher scores reflecting a positive attitude toward breastfeeding. Total IIFAS scores can be further categorized as positive to breastfeeding (IIFAS score 70–85), neutral (IIFAS score 49–69), and positive to formula feeding (IIFAS score 17–48) (Gizaw et al. 2022).

The BAS was developed to assess nurses’ and midwives’ attitudes toward breastfeeding in Sweden (Ekström et al. 2005). The instrument uses a fixed 4‐point response that includes *disagree completely* (1)*, disagree* (2)*, agree somewhat* (3), and *agree completely* (4). The mean score of all the questions results in the instrument potentially ranging from 1 to 4, where higher scores denote a more positive attitude toward breastfeeding. The questionnaire is also designed to capture diverse aspects of breastfeeding attitudes and is organized into four factors. The regulating factor consists of 10 items that center on health care personnel's orientation toward managing mothers' breastfeeding, encompassing statements related to advising and scheduling feeding. The facilitating factor, composed of nine items, explores health care personnel's support for mothers in managing their own breastfeeding, including statements on evidence‐based practices and overall breastfeeding support. The disempowering factor, comprising seven items, investigates the tendency of health care personnel to provide professional advice without considering the individual needs of the mothers seeking guidance. The Antipathy factor consists of nine items that assess health care personnel's insufficient basic breastfeeding knowledge and their negative reactions toward breastfeeding (Mäkelä et al. 2022). Cronbach's alpha reliability coefficient has been reported to be 0.51 for the BAS questionnaire, with the factor scores ranging from 0.29 to 0.80 (Ekström et al. 2005). Despite reliability limitations, the BAS instrument was selected because it directly measures medical personnel's attitudes toward breastfeeding (Mäkelä et al. 2022).

The BSES‐SF is a survey tool for measuring breastfeeding self‐efficacy, which is a woman's sense of confidence related to her perception of the benefits of breastfeeding and her ability to initiate and maintain breastfeeding (Dennis 2003). The “short form” of the tool, containing 14 items, was developed from the original Breastfeeding Self‐Efficacy Scale (BSES), which contained 33 items (Dennis and Faux 1999). For each of the 14 statements, participants select responses on a 5‐point Likert‐type scale ranging from “1” (*not very confident*) to “5” (*very confident*) to indicate their level of agreement. Scores can range from 14 to 70, with higher scores indicating greater perceived breastfeeding self‐efficacy (Dennis 2003).

The research team, which consisted of registered dietitian nutritionists, neurologists, and academics with experience conducting surveys, evaluated survey items for content validity. Based on the review's comments, the team revised the surveys for item clarity. The team also tested the usability and technical functionality of the surveys on REDCap before launching it.

### 2.3 Consent

Embedded at the beginning of each electronic survey, participants were provided a cover letter containing an invitation to participate in the research, an overview and description of the study, the risks and benefits of participating, and instructions for opting out of study participation. The invitation highlighted the anonymous and voluntary nature of the study. Completing the survey in its entirety represented participants’ consent to participate in the study.

### 2.4 Survey Administration

Both surveys could be completed in less than 10 min in consideration of minimizing participant burden, especially when surveying health care personnel (Jepson et al. 2005), such as physicians (Cunningham et al. 2015). The Bf MoMS HP—Survey 1 electronic survey remained active for a 12‐week period from July 2023 to October 2023 for health care personnel to complete. The Bf MoMS PP—Survey 2 was made available from September 2023 to December 2023, spanning a 16‐week period during which women with MS were invited to participate and provide their responses. Measures to prevent multiple participation were not fully implemented, aside from the system's allowance for participants to complete the survey at a later time if needed.

### 2.5 Participant Recruitment

Health care personnel at UIHC were invited to complete the anonymous electronic REDCap Bf MoMS HP—Survey 1. The REDCap survey link and an informational study poster were sent electronically to all UIHC health care employees through a preexisting UIHC email listserv consisting of 45,000–50,000 unique individual emails (Table S1). Reminders about the study were sent five times to each email address in the preexisting UIHC email listserv when the REDCap survey was open. Study posters that included the survey QR code and link were provided to the various department clinics, as well as displayed on televisions throughout the nursing department, inpatient hospital floors, and the university‐affiliated College of Nursing (Figure S1). In addition, the survey link and study poster were emailed to department chairs in the neurology, obstetrics, pediatrics, neonatology, and family medicine clinics to encourage their health care personnel staff to complete the survey.

The inclusion criteria to complete the Bf MoMS HP—Survey 1 were (a) age 18 years or older; (b) health care personnel or student at UIHC; and (c) providing, able to provide, or learning to provide care to postpartum women with MS. Physicians, fellows/residents, physician associates, nurse practitioners (NPs), nurses, midwives, medical students, nursing staff, medical assistants, nursing students, and other health care providers were encouraged to complete the survey. Recruiting was targeted towards the neurology, obstetrics, pediatrics, neonatology, and family medicine specialties, as these were locations where women with MS were most likely to be seen.

Women with MS were invited to complete the anonymous electronic REDCap Bf MoMS PP—Survey 2. A convenience sample approach was used to send the REDCap survey link and the informational study poster (Figure S2) electronically through a preexisting MS‐specific research email listserv, which consisted of around 3,000 unique emails from individuals who previously consented to receive emails related to research recruitment. Reminders about the study were sent three times to each email address in the email listserv during the study period.

The inclusion criteria for the Bf MoMS PP—Survey 2 were: (a) aged 18 years or older; (b) identify as a woman; (c) self‐reported physician diagnosis of MS; and (d) currently breastfeeding or previously breastfed one or more infants, regardless of method or duration. Recruitment was targeted toward women who currently or previously have breastfed.

### 2.6 Statistical Analysis

Data were checked for accuracy and possible entry errors in each survey separately. Participants with missing data for characteristics, as well as IIFAS‐related or BSES‐SF‐related questions, were excluded from the final analysis to ensure accurate instrument calculation scoring. To increase the subgroup sample size, the Bf MoMS HP—Survey 1 reclassified medical personnel into distinct categories: physicians (including physicians, and fellow/resident physicians), nurse/NPs (comprising nurses and nurse practitioners (NP)), students (encompassing medical students and nursing students), and other (including physician associates, medical assistants, and other roles). Both survey participants’ characteristic information was reported as counts and percentages of the reclassified categories.

The total scores for the IIFAS and BSES‐SF were calculated by adding the scores of all the questions to obtain the overall mean scores and standard deviation. The total scores for BAS were calculated by obtaining the overall mean score of all questions, as well as the mean scores of the specific BAS factors questions. Subsequently, the mean scores of the instruments were stratified by the demographic categories. One‐way analysis of variance (ANOVA) was used to assess differences between the demographic categories. Sensitivity analysis to assess the association between mean IIFAS scores, and BAS total and factor scores for the Bf MoMS HP—Survey 1, as well as between mean age, years of MS diagnosis, IIFAS scores and BSES‐SF scores for the Bf MoMS PP survey 2, was conducted using Pearson's correlation. *p* values of  ≤ 0.05 were considered statistically significant.

All data analyses were performed using SAS software (version 9.4, SAS Institute, Inc.).

## 3 Results

In the Bf MoMS HP—Survey 1, a total of 139 individuals responded; of these, 100 completed the entire survey for a response rate of 71.9%. Surveys with missing question responses (n = 8) were removed for a final sample size of 92 participants used for analysis (Figure S3). Analysis of recruitment email engagement revealed that a range of 30.6% to 33.7% of recipients opened their emails at least once (Table S1).

For the Bf MoMS PP—Survey 2, a total of 310 survey responses were initially received, and of these, 198 completed the survey for a response rate of 63.9% (Figure S4). Other survey responses were further excluded for participants living outside the United States (*n* = 28) and identifying sex as male (*n* = 1; Figure S4).

Of the final Bf MoMS HP—Survey 1 sample (*n* = 92), 46.7% nurses or nurse practitioners (*n* = 43), 23.9% pediatrics (*n* = 22), and 39.1% of the sample reported 0–5 years of service (*n* = 36; Table 1). Scores from the IIFAS Bf MoMS HP—Survey 1 revealed that 68.5% had an overall neutral attitude (*n* = 63; Table 1). As assessed by mean IIFAS scores, attitudes towards breastfeeding significantly differed by health care profession (p = 0.04), with physicians and nurses/NPs having the highest IIFAS scores (67.6 ± 6.72 and 67.6 ± 9.60, respectively), although a neutral attitude. Additionally, IIFAS mean scores differed between medical specialties (*p* = 0.01), with neonatology (69.8 ± 8.89) having positive attitudes towards breastfeeding. IIFAS mean scores differed by years of service in health care (*p* = 0.02), with respondents with 16 or more years of service having positive attitudes toward breastfeeding (70.56 ± 9.78). In contrast, no statistically significant differences were observed for sex, health care profession, medical specialty, or years of service among the mean BAS total and factor scores when stratified by the characteristics of the participants (Table S2).

**TABLE 1:** Bf MoMS HP—Survey 1 participant characteristics and IIFAS scores.

	Survey respondents		IIFAS		
Characteristics	Frequency	%	Mean (std dev)	Interpretation	*p* value^a^
All participants	**92**		65.8 (8.47)	Neutral BF	
Sex					0.27
Male	7	7.61	63.0 (3.61)	Neutral BF	
Female	83	90.2	65.81 (8.73)	Neutral BF	
Other	2	2.17	74.0 (1.41)	Positive BF	
Profession					**0.04**
Physician	17	18.5	67.6 (6.72)	Neutral BF	
Nurse/NP	43	46.7	67.6 (9.60)	Neutral BF	
Health sciences student	7	7.61	62.8 (7.07)	Neutral BF	
Other	25	27.2	60.9 (4.74)	Neutral BF	
Health care specialty					**0.01**
Neurology	5	5.43	62.4 (10.3)	Neutral BF	
Obstetrics	20	21.7	68.3 (8.12)	Neutral BF	
Pediatrics	22	23.9	63.7 (8.48)	Neutral BF	
Neonatology	10	10.9	69.8 (8.89)	Positive BF	
Family medicine	12	13.0	66.9 (6.02)	Neutral BF	
Health sciences student	10	10.9	58.2 (3.94)	Neutral BF	
Other	13	14.1	68.5 (8.99)	Neutral BF	
Years of service (years)					**0.01**
0–5	36	39.1	64.3 (6.64)	Neutral BF	
6–10	20	21.7	65.1 (9.26)	Neutral BF	
11–15	5	5.43	68.6 (10.9)	Neutral BF	
16+	21	22.8	70.9 (9.30)	Positive BF	
Student	10	10.9	60.2 (4.34)	Neutral BF	
IIFAS					
Positive breastfeeding attitude	28	30.4			
Neutral breastfeeding attitude	63	68.5			
Positive formula feeding attitude	1	1.09			
Total BAS score	92	100			

Abbreviations: BAS, breastfeeding attitude scale; BF, breastfeeding; IIFAS, Iowa Infant Feeding Scale; NP, nurse practitioner.

^a^ *p* value determined by one‐way ANOVA.

Of the final Bf MoMS PP—Survey 2 sample (*n* = 169), most participants reported having been diagnosed with RRMS (85.2%), identified their ethnicity as White (82.8%), being married (34.9%), full‐time employed (83.4%), exclusively breastfeeding their children for the first 6 months (63.9%), and did not use DMTs while breastfeeding (88.8%; Table 2). There were statistically significant differences in mean Bf MoMS PP—Survey 2 IIFAS scores (*p* = 0.001) by race of the respondent, with women identifying as Middle Eastern/North African and Latino having positive attitudes toward breastfeeding (78.0 ± 5.66 and 74.3 ± 6.00, respectively). IIFAS mean scores further differed (*p* < 0.001) based on having exclusively breastfed for 6 months, with women who reported exclusively breastfeeding for 6 months having positive attitudes towards breastfeeding (70.1 ± 7.17). The BSES‐SF mean scores varied by age (*p* = 0.03), with mean scores increasing with the increasing age of the respondent, with the 60+ age range scoring highest (53.0 ± 4.02). Similarly, increased self‐efficacy mean scores were reported with a higher number of children (*p* = 0.01); women with 1 child had the lowest mean BSES‐SF scores (47.1 ± 9.38), while women with 3+ children had the highest (51.7 ± 5.26; Table 3). The total number of children breastfed also had varied self‐efficacy scores (*p* = 0.01), with women who breastfed 3+ children having the highest (51.7 ± 5.26). Along with this, women who exclusively breastfed for 6 months had the highest BSES‐SF mean scores (52.2 ± 4.93; *p* < 0.001).

**TABLE 2:** Bf MoMS PP—Survey 2 Participant characteristics, IIFAS, and BSES‐SF mean scores.

	Survey respondents		IIFAS			BSES‐SF	
Characteristics	Frequency or mean (std dev)	%	Mean (std dev)	Interpretation	*p* value^a^	Mean (std dev)	*p* value^a^
All Participants	169		68.86 (7.32)	Neutral		49.68 (7.42)	
Age	49.4 (11.1)				0.46		**0.03**
< 40	42	24.9	69.0 (7.78)	Neutral		47.9 (6.85)	
40–49	53	31.4	67.6 (6.99)	Neutral		49.2 (7.06)	
50–59	43	25.4	70.0 (7.65)	Positive		49.7 (9.44)	
60+	31	18.3	69.1 (6.81)	Neutral		53.0 (4.02)	
MS diagnosis					1.00		0.35
CIS	4	2.37	69.5 (7.59)	Neutral		47.3 (10.6)	
RRMS	144	85.2	68.8 (7.38)	Neutral		49.5 (7.48)	
PPMS	8	4.73	69.0 (10.6)	Neutral		48.5 (8.67)	
SPMS	11	6.51	69.5 (4.37)	Neutral		53.6 (3.50)	
Other	2	1.18	68.5 (7.78)	Neutral		53.5 (2.12)	
Years of MS diagnosis	14.0 (10.8)				0.17		
< 5	45	26.6	68.6 (7.75)	Neutral		47.5 (9.23)	
5–9	37	21.9	68.9 (7.11)	Neutral		49.1(7.19)	
10–14	27	16.0	66.1 (6.71)	Neutral		48.1 (6.25)	
15–19	20	11.8	70.5 (7.07)	Positive		53.0 (3.64)	
20+	40	23.7	70.2 (7.31)	Positive		52.0 (6.50)	
Medication use during breastfeeding					0.11		0.58
Yes	17	10.2	66.2 (7.40)	Neutral		48.7 (7.15)	
No	150	89.8	69.2 (7.23)	Neutral		49.8 (7.49)	
Marital status					0.59		0.23
Married	141	83.4	68.7 (7.17)	Neutral		49.2 (7.63)	
Single	6	3.55	68.2 (10.7)	Neutral		50.5 (8.76)	
Divorced	14	8.28	70.0 (7.73)	Positive		53.5 (4.13)	
Widowed	2	1.18	63.0 (8.48)	Neutral		54.0 (2.83)	
Domestic partnership	4	2.37	72.5 (6.24)	Positive		53.3 (3.77)	
Other	2	1.18	74.0 (8.49)	Positive		45.0 (8.49)	
Race					**0.01**		0.26
Black	7	4.14	62.7 (6.07)	Neutral		45.7 (8.98)	
Latino	10	5.92	74.3 (6.00)	Positive		53.2 (4.26)	
Middle Eastern/North African	2	1.18	78.0 (5.66)	Positive		52.5 (3.54)	
White	140	82.8	68.8 (7.18)	Neutral		49.7 (7.20)	
Prefer Not to Answer	4	2.37	68.0 (5.60)	Neutral		52.5 (3.79)	
Other race	6	3.55	65.7 (8.57)	Neutral		46.2 (13.9)	
Employment status					0.30		0.52
Full‐time	59	34.9	67.7 (7.10)	Neutral		48.6 (8.66)	
Part‐time	24	14.2	70.0 (7.16)	Positive		50.0 (7.58)	
Self‐employment	12	7.10	70.9 (7.48)	Positive		49.0 (8.47)	
Unemployed/homemaker	49	29.0	68.4 (7.70)	Neutral		50.4 (5.90)	
Student	2	1.18	64.0 (4.24)	Neutral		44.5 (7.78)	
Other	23	13.6	71.0 (7.08)	Positive		51.4 (6.08)	
Number of children					0.31		**0.01**
1	37	21.9	69.0 (8.15)	Neutral		47.1 (9.38)	
2	82	48.5	68.1 (7.56)	Neutral		49.6 (7.25)	
3+	50	29.6	70.1 (6.17)	Positive		51.7 (5.26)	
Infants breastfed exclusively 6 months					**0.0004**		**< 0.0001**
Exclusive	108	63.9	70.1 (7.17)	Positive		52.2 (4.93)	
Partial	28	16.6	69.5 (6.23)	Neutral		50.1 (6.52)	
Never	33	19.5	64.4 (7.16)	Neutral		41.1 (8.61)	
Total number of infant(s) breastfed					0.06		**0.01**
1	37	21.9	67.1 (8.27)	Neutral		47.1 (9.38)	
2	82	48.5	69.7 (6.85)	Neutral		49.6 (7.25)	
3+	50	29.6	64.0 (2.65)	Neutral		51.7 (5.26)	

Abbreviations: BSES‐SF, Breastfeeding Self‐efficacy Scale‐Short Form; IIFAS, Iowa Infant Feeding Attitudes Scale; MS, multiple sclerosis; std dev, standard deviation.

^a^ *p* value determined by one‐way ANOVA.

**TABLE 3:** Bf MoMS PP—Survey 2 Pearson's correlation between study participant's age and years of MS diagnosis with IIFAS and BSES—SF scores.

	Age	MS years	IIFAS	BSES—SF
Age	1	0.61 < 0.0001	0.09 0.26	0.23 0.002
MS years		1	0.10 0.22	0.23 0.003
IIFAS			1	0.40 < 0.0001
BSES—SF				1

For the Bf MoMS HP—Survey 1, there were associations between IIFAS mean scores and BAS total and factor scores when analyzed using Pearson's correlation coefficients (Table 3). The mean IIFAS scores were negatively correlated with BAS Total score (*r* = −0.36, *p* = 0.0005), BAS–Regulating (*r* = −0.51, *p* < 0.0001), and BAS–Disempowering (*r* = −0.48, *p* < 0.0001), while there was a positive correlation with BAS–Facilitating (*r* = 0.33, *p* = 0.002). Conversely, mean BAS total scores showed positive correlations with BAS–Regulating (*r* = 0.71, *p *< 0.0001), BAS–Facilitating (*r* = 0.30, *p* = 0.004), BAS–Disempowering (*r* = 0.80, *p* < 0.0001), and BAS–Antipathy factors (*r* = 0.34, *p* = 0.0008).

## 4 Discussion

The present cross‐sectional surveys revealed differences among health care personnel's attitudes towards breastfeeding in women with MS, as well as variations in infant feeding attitudes and breastfeeding self‐efficacy among women with MS who had breastfeeding experience. Health care personnel with extensive experience, particularly those with 16 or more years and specialized in neonatology, exhibited positive attitudes. Among women with MS who are currently breastfeeding or previously breastfed, race and exclusively breastfeeding for 6 months were found to be positively associated with the infant feeding attitudes. Results also suggest that multiparity, having exclusively breastfed for 6 months, and older age were associated with increased breastfeeding self‐efficacy among women with MS. Given the variation in attitudes among health care personnel, differences regarding breastfeeding counseling and support may further impact attitudes towards breastfeeding among women with MS; thus, the clinical implication addressing these differences through educational interventions for lactation for health care personnel, as well as educational resources tailored for women with MS should be considered, may enhance attitudes and support for breastfeeding.

Health care professionals with over 16 years of service demonstrated positive attitudes toward breastfeeding, possibly stemming from personal experiences such as their own breastfeeding or having a partner who breastfed. A study involving pediatric nurses found that those with personal breastfeeding experiences exhibited significantly higher knowledge and attitude scores for breastfeeding (Brewer 2012). Similarly, a large national study of physicians highlighted that previous personal or spousal breastfeeding experiences were the greatest predictors of physicians' self‐confidence in counseling patients (Freed 1995). Thus, personal experiences with breastfeeding can potentially contribute to a more favorable perspective on breastfeeding and influence health care personnel's attitudes and advocacy of breastfeeding to patients. To address health care personnel with fewer years of service whose attitudes towards breastfeeding may be neutral, continued education or training on the topic of breastfeeding should be considered (Meek et al. 2020; Holmes et al. 2012).

Neurology, as a medical specialty, exhibited the lowest mean scores and neutral attitudes towards breastfeeding in the present study, in contrast to neonatology, which revealed the highest mean scores and positive attitudes towards breastfeeding. This difference in attitudes may stem from neonatologists' understanding of the need for premature and/or ill infants to have breast milk (Altobelli et al. 2020). Opportunities for ongoing lactation education to achieve proficiency in supporting lactation and breastfeeding have also been emphasized by the Surgeon General's Call to Action to Support Breastfeeding in 2011 for clinicians caring for women and children (Office of the Surgeon General 2011). While neurologists may not provide care on a daily basis to women and children, potential clinical implications may include ongoing education or training opportunities on breastfeeding, which may positively influence attitudes toward breastfeeding; however, further research is necessary to better factors contributing to the neutral attitudes expressed by personnel in the field of neurology.

IIFAS and BSES‐SF scores were higher among women with MS who exclusively breastfed their children for 6 months, reflecting more positive attitudes and higher self‐efficacy regarding breastfeeding. A systematic review conducted in 2020 of 5 cohort studies and 11 cross‐sectional studies found that self‐efficacy and attitudes towards breastfeeding were among the strongest predictors of exclusive breastfeeding for 6 months (Wu et al. 2022). The systematic review further revealed that women with higher breastfeeding self‐efficacy were more likely to breastfeed exclusively for 6 months (Wu et al. 2022); however, this review did not specifically investigate the impact among individuals with chronic illnesses, such as MS. Current theories regarding the relationship between self‐efficacy and breastfeeding suggest that psychosocial factors, such as professional and partner support, are also predictors to initiate breastfeeding (Meedya et al. 2010; Henshaw et al. 2015). Women with higher self‐efficacy, which is based on social cognitive theory's (Bandura 1977) perceived ability to perform a specific task or behavior, are more likely to persist through challenges related to breastfeeding with self‐encouraging thoughts and reflect more positively after overcoming breastfeeding challenges (de Jager et al. 2014; James et al. 2020; Henshaw et al. 2015). Previous studies also corroborate the relationship between exclusively breastfeeding for 6 months and increasing positive attitudes toward breastfeeding (de Jager et al. 2014; Cernadas et al. 2003). One study found as high as a seven‐fold increase in rates of exclusive breastfeeding at 6 months in women with “very good” versus “fair” self‐reported attitudes towards breastfeeding (Cernadas et al. 2003). Overcoming breastfeeding challenges, potentially with the help of health care personnel, can positively reaffirm belief in one's ability to both persist and breastfeed exclusively for 6 months (de Jager et al. 2014).

Higher breastfeeding self‐efficacy was observed among older women with MS, particularly those aged 60 years and above. This finding aligns with a previous study observing a positive relationship between age and breastfeeding self‐efficacy among postpartum women (Mercan and Tari Selcuk 2021); however, it contrasts with other studies indicating that maternal age has little to no effect on breastfeeding self‐efficacy, with none of the previous studies assessing women with chronic illness, let alone MS (Amini et al. 2019; Dennis 2006). It is also important to note that the present study recruited women who were currently or had previously breastfed, without further differentiation based on timing (i.e., current or past), which could have influenced participants' responses to the BSES‐SF questionnaire, given the unlikelihood of women breastfeeding at age 60 years or above. Moreover, the observed association between older age and greater breastfeeding self‐efficacy may be confounded by other situational and demographic factors not assessed in the present survey, such as the past availability of certain DMTs, breastfeeding history, available support, and aspects of psychological well‐being (Eslami et al. 2020; Lawal and Idemudia 2017). While the present findings suggest that older age increases breastfeeding self‐efficacy in women, further research is warranted, especially differentiating between current or previous breastfeeding within the context of diagnosed chronic illnesses, such as MS.

Multiparous women with MS had greater perceived self‐efficacy in breastfeeding compared to women with MS with one child. This is corroborated by findings from studies in the general population, where having multiple children is associated with higher self‐efficacy levels. For instance, one study found that first‐time mothers exhibited significantly lower self‐efficacy scores at 1 week and 8 weeks postpartum compared to multiparous women (de Jager et al. 2014). Furthermore, longitudinal studies indicate that breastfeeding self‐efficacy scores tend to rise over the course of 4 months postpartum, particularly among multiparous women who start with a higher baseline self‐efficacy (Dennis 2006; Semenic et al. 2008). However, conflicting evidence exists, with some international studies suggesting that multiparity does not correlate with elevated self‐efficacy (Amini et al. 2019), though none were evaluated specifically among women with MS. Therefore, the positive relationship observed in the study between increased parity and greater breastfeeding self‐efficacy among women with MS may reflect their accumulated experience and knowledge in breastfeeding. This can potentially be explained by a mother's initial breastfeeding experience, which greatly influences her future perceptions of breastfeeding (Mulder and Johnson 2010). A previous study found that mothers with no prior experience or unsuccessful breastfeeding attempts are more likely to develop negative perceptions compared to those with positive experiences, and notably, positive breastfeeding experiences are associated with increased breastfeeding self‐efficacy (Mulder and Johnson 2010). Prior breastfeeding satisfaction has been strongly correlated with the likelihood of breastfeeding subsequent children (Schafer et al. 2017). Educational interventions, such as informational‐motivational‐behavioral skills programs, have been shown to enhance breastfeeding self‐efficacy among first‐time mothers (Wu et al. 2022), suggesting that education and counseling on breastfeeding positioning as well as techniques specially tailored for women with MS may be beneficial to increase breastfeeding self‐efficacy.

This study's strengths include using anonymous survey methodologies, enhancing disclosure of sensitive information (Murdoch et al. 2014), and validated questionnaires; however, there are several limitations. While anonymity promotes openness, it complicates identifying the population, understanding responses, and eliminating duplicates (Andrade 2020). The Bf MoMS HP—Survey 1 data from a single hospital limits generalizability, and the high “other” profession characteristics category suggests adding professions like pharmacists and dietitians as answer options in future studies. The Bf MoMS PP—Survey 2 lacked data on time since the last breastfeeding event, increasing recall bias risk. Both surveys had small, diverse samples, possibly limiting representation of health care personnel and women with MS, and may reflect volunteer and nonresponse biases due to time constraints or stigma (Alvarez and Vanbeselaere 2005). Most importantly, further research is needed to assess breastfeeding benefits for women with MS, understand health care personnel's influence on breastfeeding, and address racial disparities to improve maternal and infant health outcomes.

## Author Contributions

**Solange M. Saxby**: conceptualization, investigation, funding acquisition, writing–original draft, methodology. **Carlyn Haas**: writing–original draft, writing–review and editing. **Anna Klein**: writing–review and editing, methodology, project administration. **Tyler J. Titcomb**: formal analysis, writing–review and editing, software. **Farnoosh Shemirani**: writing–review and editing. **Terry Wahls**: writing–review and editing, resources, supervision. **Linda Snetselaar**: writing–review and editing, resources, supervision. **Christine Gill**: funding acquisition, writing–review and editing, supervision, methodology. **Pamela Mulder**: supervision, conceptualization, investigation, funding acquisition, writing–review and editing, methodology, resources.

## Ethics Statement

The study protocol and tools were reviewed and approved by the University of Iowa Institutional Review Board (IRB# 202305124) prior to the study commencing.

## Conflicts of Interest

Terry Wahls personally follows and promotes the Wahls diet. She has an equity interest in the following companies: Terry Wahls LLC, TZ Press LLC, The Wahls Institute, PLC, FBB Biomed Inc., Levels Health Inc., Foogal Inc., and the website http://www.terrywahls.com. She also owns the copyright to the books Minding My Mitochondria (2nd Edition) and The Wahls Protocol, The Wahls Protocol Cooking for Life, and the trademarks The Wahls Protocol and Wahls diet, Wahls Paleo diet, and Wahls Paleo Plus diets, and Wahls Behavior Change. She has completed grant funding from the National Multiple Sclerosis Society for the Dietary Approaches to Treating Multiple Sclerosis Related Fatigue Study. She has financial relationships with Vibrant America LLC, Standard Process Inc., MasterHealth Technologies Inc., Foogal Inc., and the Institute for Functional Medicine Inc. She receives royalty payments from Penguin Random House. Wahls has conflict‐of‐interest management plans in place with the University of Iowa and the Iowa City Veteran's Affairs Medical Center. The other authors declare no conflicts of interest.

### Peer Review

The peer review history for this article is available at https://publons.com/publon/10.1002/brb3.70468

## Supporting information



Supporting Information

## Data Availability

The data presented in this study are available upon reasonable request from the corresponding author.
