# Supplementary material for: Assessing Breastfeeding Attitudes and Self‐Efficacy Among Health care Personnel and Women With Multiple Sclerosis: Two Cross‐Sectional Surveys
Source: Brain Behav. 2025 Apr 18;15(4):e70468. doi: 10.1002/brb3.70468 (PMC12007018; doi:10.1002/brb3.70468)
Supplement: Supplementary file 1 — Supporting Information [file BRB3-15-e70468-s001.docx]

**SUPPLEMENTAL MATERIALS**

| **Table S1. Recruitment email dates, sent emails, and opened messages for the University of Iowa email list serve for Bf MoMS HP – Survey 1** | | | |
| --- | --- | --- | --- |
| **Date Sent** | **Email Messages Sent (n)** | **E-mail Messages Open (n)** | **Percent E-mail Messages Open (%)** |
| 07/17/23 | 45,509 | 14,098 | 31.0 |
| 08/07/23 | 45,459 | 14,126 | 31.1 |
| 09/04/23 | 45,431 | 15,305 | 33.7 |
| 09/22/23 | 50,291 | 16,370 | 32.6 |
| 10/02/23 | 50,335 | 16,015 | 31.8 |

| **Table S2. Bf MoMS HP – Survey 1 BAS subscore means and standard deviation stratified by demographics** | | | | | |
| --- | --- | --- | --- | --- | --- |
|  | **BAS -**  **Total** | **BAS -Regulating** | **BAS -Facilitating** | **BAS -Disempowering** | **BAS -**  **Antipathy** |
| All Participants | 2.54 (0.16) | 2.46 (0.29) | 2.67 (0.25) | 2.25 (0.42) | 2.74 (0.21) |
| Sex |  |  |  |  |  |
| Male | 2.54 (0.16) | 2.54 (0.11) | 2.56 (0.22) | 2.21 (0.48) | 2.77 (0.28) |
| Female | 2.55 (0.17) | 2.45 (0.31) | 2.67 (0.25) | 2.26 (0.41) | 2.74 (0.20) |
| Other | 2.45 (0.03) | 2.50 (0.14) | 2.89 (0.00) | 1.75 (0.12) | 2.39 (0.15) |
| p-value^a^ | 0.70 | 0.70 | 0.22 | 0.23 | 0.06 |
| Medical Personnel |  |  |  |  |  |
| Physician | 2.46 (0.09) | 2.38 (0.22) | 2.69 (0.22) | 2.04 (0.25) | 2.63 (0.16) |
| Nurse/NP | 2.54 (0.17) | 2.41 (0.32) | 2.70 (0.26) | 2.27 (0.43) | 2.74 (0.22) |
| Other | 2.59 (0.18) | 2.57 (0.29) | 2.60 (0.25) | 2.32 (0.44) | 2.80 (0.22) |
| Student | 2.58 (0.16) | 2.52 (0.23) | 2.62 (0.14) | 2.36 (0.51) | 2.77 (0.17) |
| p-value^a^ | 0.10 | 0.10 | 0.41 | 0.13 | 0.09 |
| Medical Specialty |  |  |  |  |  |
| Neurology | 2.54 (0.29) | 2.54 (0.40) | 2.71 (0.23) | 1.99 (0.60) | 2.78 (0.27) |
| Obstetrics | 2.51 (0.12) | 2.43 (0.25) | 2.64 (0.20) | 2.24 (0.36) | 2.70 (0.19) |
| Pediatrics | 2.59 (0.19) | 2.56 (0.30) | 2.69 (0.20) | 2.33(0.48) | 2.71 (0.21) |
| Neonatology | 2.52 (0.18) | 2.30 (0.37) | 2.74 (0.36) | 2.21 (0.37) | 2.78 (0.24) |
| Family Medicine | 2.53 (0.13) | 2.47 (0.26) | 2.61 (0.26) | 2.29 (0.41) | 2.71 (0.19) |
| Student | 2.52 (0.16) | 2.48 (0.26) | 2.54 (0.22) | 2.28 (0.44) | 2.74 (0.23) |
| Other | 2.55 (0.15) | 2.38 (0.29) | 2.74 (0.29) | 2.19 (0.38) | 2.82 (0.21) |
| p-value^a^ | 0.83 | 0.33 | 0.42 | 0.79 | 0.73 |
| Years of Service |  |  |  |  |  |
| 0-5 | 2.57 (0.18) | 2.51 (0.32) | 2.65 (0.21) | 2.34 (0.47) | 2.72 (0.20) |
| 6-10 | 2.54 (0.15) | 2.43 (0.29) | 2.65 (0.29) | 2.26 (0.33) | 2.75 (0.19) |
| 11-15 | 2.53 (0.19) | 2.42 (0.15) | 2.78 (0.34) | 2.07 (0.38) | 2.73 (0.24) |
| 16+ | 2.52 (0.14) | 2.39 (0.32) | 2.73 (0.25) | 2.10 (0.37) | 2.75 (0.24) |
| Student | 2.52 (0.15) | 2.45(0.21) | 2.55 (0.20) | 2.30 (0.45) | 2.75 (0.24) |
| p-value^a^ | 0.82 | 0.66 | 0.29 | 0.26 | 0.98 |
| Abbreviations: BAS, Breastfeeding Attitude Scale.  ^a^ p-value determined by 1-way ANOVA. | | | | | |

| **Table S3. Bf MoMS HP – Survey 1 Pearson’s Correlation between IIFAS mean scores and BAS mean subscore** | | | | | | |
| --- | --- | --- | --- | --- | --- | --- |
|  | **IIFAS** | **BAS -**  **Total** | **BAS -Regulating** | **BAS -Facilitating** | **BAS -Disempowering** | **BAS -Antipathy** |
| **IIFAS** | **1** | -0.36  (0.0005) | -0.51  (<0.0001) | 0.33  (0.002) | -0.48  (<0.0001) | 0.03  (0.82) |
| **BAS - Total** |  | **1** | 0.71  (<0.0001) | 0.30  (0.004) | 0.80  (<0.0001) | 0.34  (0.0008) |
| **BAS - Regulating** |  |  | **1** | -0.25  (0.01) | 0.59  (<0.0001) | -0.03  (0.78) |
| **BAS - Facilitating** |  |  |  | **1** | -0.01  (0.92) | 0.08  (0.45) |
| **BAS - Disempowering** |  |  |  |  | **1** | 0.03  (0.76) |
| **BAS - Antipathy** |  |  |  |  |  | **1** |

**Figure S1. Bf MoMS HP – Survey 1 Study recruitment poster**


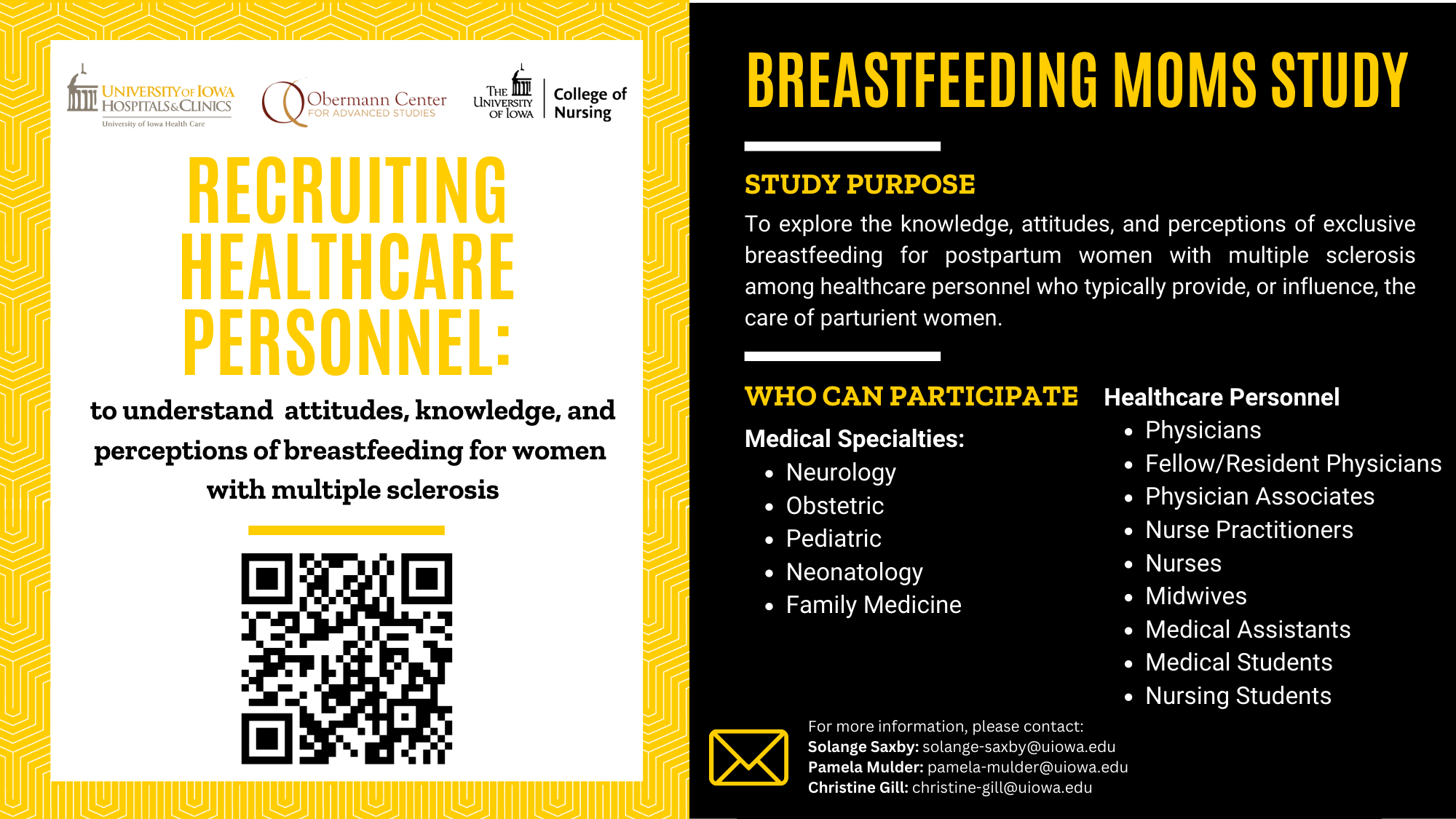


**Figure S2. Bf MoMS PP – Survey 2 Study recruitment poster**


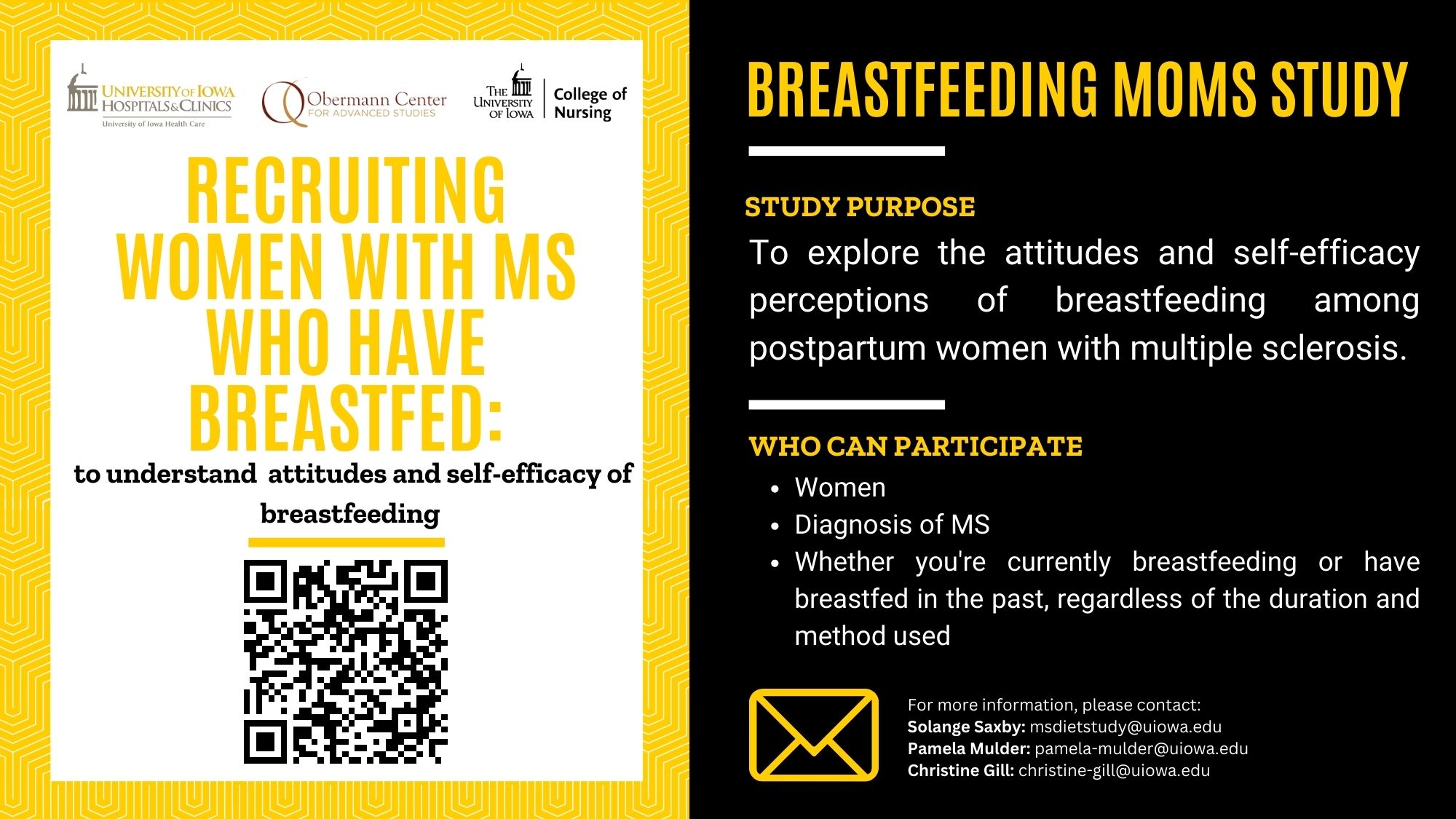


**Figure S3. Bf MoMS HP – Survey 1 Flowchart for the inclusion process for completed participant surveys**


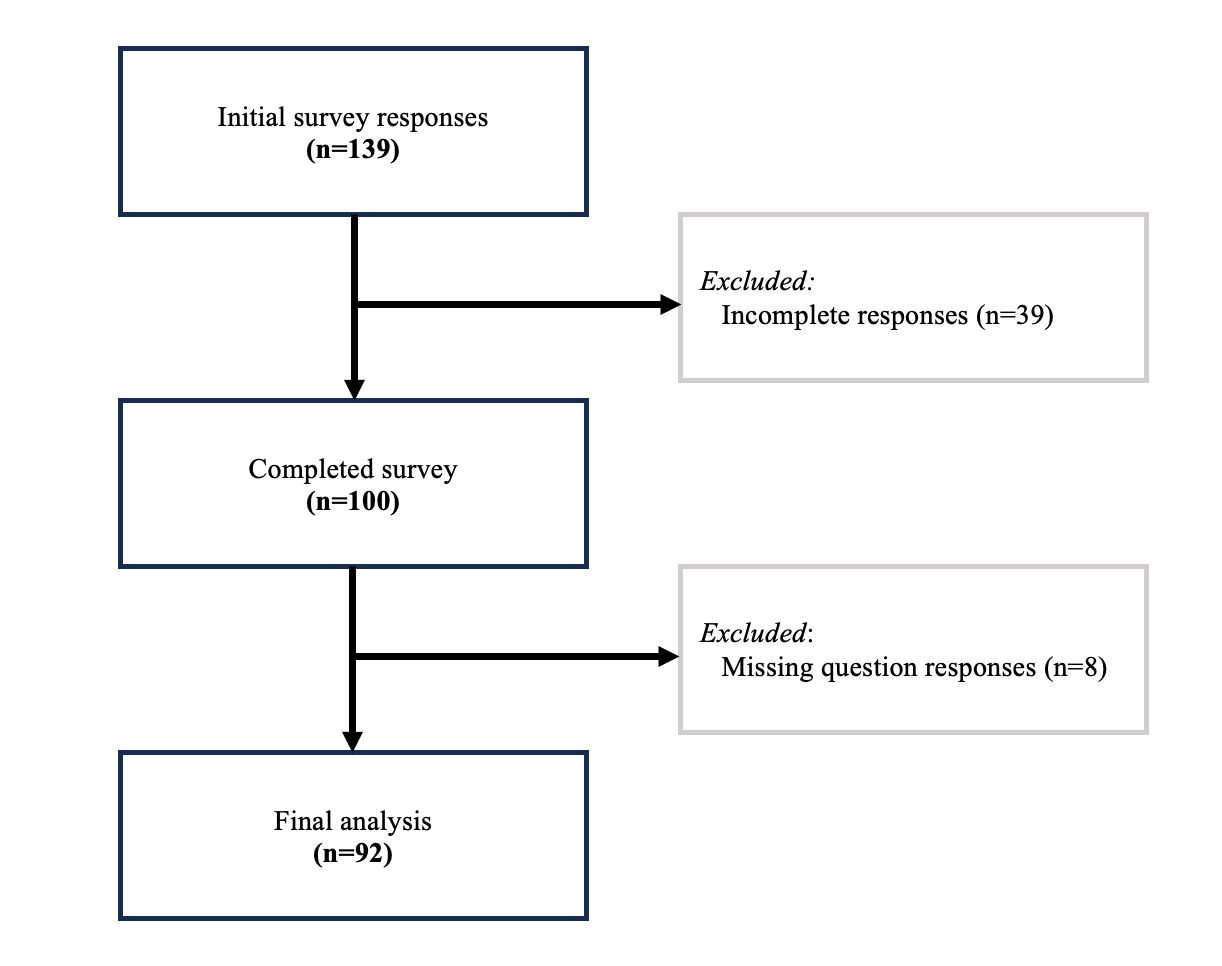


**Figure S4. Bf MoMS PP – Survey 2 Flowchart for the inclusion process for completed participant surveys.**

Initial survey responses

(n = 310)

Completed survey

(n = 198)

*Excluded:*

Incomplete responses

(n = 102)

*Excluded:*

Outside of the USA

(n = 28)

Participants within the USA

(n = 170)

Final Analysis

(n = 169)

*Excluded:*

Male

(n = 1)
